# Rechallenge of immunotherapy beyond progression in patients with extensive-stage small-cell lung cancer

**DOI:** 10.3389/fphar.2022.967559

**Published:** 2022-09-06

**Authors:** Lingling Li, Tingting Liu, Qingyan Liu, Shuai Mu, Haitao Tao, Xuhui Yang, Yao Li, Qi Xiong, Lijie Wang, Yi Hu

**Affiliations:** ^1^ School of Medicine, Nankai University, Tianjin, China; ^2^ Senior Department of Oncology, The Fifth Medical Center of Chinese PLA General Hospital, Beijing, China; ^3^ Department of Oncology, Chinese PLA General Hospital, Beijing, China

**Keywords:** small-cell lung cancer, immune checkpoint inhibitor, PD-1/PD-L1 inhibitors, prognosis, rechallenge of immunotherapy

## Abstract

**Background:** Rechallenge of immunotherapy beyond progression (RIBP) has been demonstrably effective in a variety of cancers. Our study aims to investigate the efficacy of RIBP in small-cell lung cancer (SCLC) patients under real-world conditions.

**Methods:** SCLC patients who experienced progressive disease after receiving programmed cell death-1 (PD-1)/programmed cell death ligand-1 (PD-L1) inhibitors combined with chemotherapy from January 2017 to October 2021 were enrolled. The study population was divided into two groups: the RIBP group and the discontinuation of immunotherapy beyond progression (DIBP) group. Inverse propensity score weighting (IPSW) method was used to balance the clinical baseline characteristics. The short-term and long-term efficacy of the two groups was compared.

**Results:** 100 SCLC patients were included in this study. There were 45 patients in the RIBP group and 55 patients in the DIBP group. The disease control rate (DCR) and the proportion of durable clinical benefit (DCB) were significantly higher in the RIBP group (DCR: 79.7% vs. 55.7%, *p* = 0.027; DCB: 40.7 vs. 20.7%, *p* = 0.025) after weighting. The median progressive-free survival (PFS) in the RIBP group was significantly longer than the DIBP group in the total population (mPFS: 4.8 vs. 2.4 months, *p* = 0.002), while there was no significant difference in overall survival (OS) of the two groups (mOS: 17.4 vs. 8.0 months, *p* = 0.098). In the weighted first-line initial immunotherapy subgroup, PFS and OS were significantly improved in the RIBP group (mPFS: 4.5 vs. 2.8 months, *p* = 0.017; mOS: 11.6 vs. 5.4 months, *p* = 0.028). After weighting, the RIBP group had a significantly longer PFS than the DIBP group in the SD/PD response to the initial immunotherapy subgroup (mPFS: 6.8 vs. 1.8 months, *p* = 0.026).

**Conclusion:** Rechallenge of PD-1/PD-L1 inhibitors could bring benefits to SCLC patients, especially in the first-line initial immunotherapy subgroup or SD/PD response to the initial immunotherapy subgroup.

## Introduction

Lung cancer is still the leading cause of cancer-related deaths in the world (Bray et al., 2018), which is mainly classified as non-small-cell lung cancer (NSCLC) and small-cell lung cancer (SCLC). The incidence of SCLC is relatively low, accounting for only about 15%. However, there were limited treatment options for SCLC patients, and most SCLC patients had poor prognosis (Oronsky et al., 2017). SCLC patients are generally classified as limited-stage SCLC (LS-SCLC) and extensive-stage SCLC (ES-SCLC). Approximately two-thirds of SCLC patients had extensive disease at diagnosis. In the past few decades, the standard first-line treatment for ES-SCLC patients was platinum-based doublet chemotherapy (Früh et al., 2013; Rudin et al., 2015). However, the median overall survival (OS) of ES-SCLC patients was only 8–13 months, with a 5-years OS rate of approximately 3% (Rossi et al., 2012; Bernhardt and Jalal, 2016).

In recent years, immune checkpoint inhibitors (ICIs), especially for programmed cell death-1 (PD-1)/programmed cell death ligand-1 (PD-L1) inhibitors, have brought new hope to SCLC patients (Facchinetti et al., 2020). Clinical trials, including Impower133 and CASPIAN, have demonstrated the first-line application of PD-L1 inhibitors could improve the survival of advanced SCLC patients (Horn et al., 2018; Paz-Ares et al., 2019). In CheckMate032 study, nivolumab monotherapy as third- or later line treatment could bring recurrent SCLC patients survival benefit (Ready et al., 2019). Based on these promising results, Food and Drug Administration (FDA) has approved the application of atezolizumab and durvalumab in first-line treatment and nivolumab in third- or later line treatment for advanced SCLC patients. Nevertheless, progression on previous anti-PD-1/PD-L1 inhibitors is inevitable, while the treatment strategy for SCLC patients beyond progression is still a challenge.

Previous study reported that some patients might benefit from rechallenge of immunotherapy beyond Response Evaluation Criteria in Solid Tumors (RECIST)1.1-defined progression (Chiou and Burotto, 2015). It was documented that rechallenge of immunotherapy beyond progression (RIBP) was effective for advanced renal cell carcinoma, squamous cell carcinoma of the head and neck, melanoma, NSCLC, and urothelial carcinoma (George et al., 2016; Escudier et al., 2017; Long et al., 2017; Gandara et al., 2018a; Beaver et al., 2018; Haddad et al., 2019; Fukuokaya et al., 2021). For instance, in a subgroup analysis of Checkmate025 study, patients with advanced renal cell carcinoma in the RIBP group had a significantly longer post-progression OS than those in the discontinuation of immunotherapy beyond progression (DIBP) group (Escudier et al., 2017). In addition, in a pooled analysis, among the melanoma patients continuing the application of PD-1 inhibitors, 19% (95/500) patients had a more than 30% decrease of tumor burden, and patients continuing the PD-1 inhibitors had an improved OS than those discontinuing the immunotherapy (Long et al., 2017; Beaver et al., 2018). OAK study also demonstrated the survival benefit of RIBP for advanced NSCLC patients (Gandara et al., 2018a). However, in a European retrospective study, there was no difference of post-progression OS between RIBP group and DIBP group in NSCLC patients with PD-L1 ≥ 50% (Metro et al., 2019). Taken together, the evidence on the effect of RIBP is limited. In Checkmate032 study, 30.6% (30/98) of the included SCLC patients continued receiving nivolumab, but the effect of the RIBP group was not reported (Antonia et al., 2016; Spagnolo et al., 2021). Thus, it remains unclear whether SCLC patients could benefit from the retreatment of immunotherapy beyond progression. Our study aims to explore whether the rechallenge of PD-1/PD-L1 inhibitors could bring benefit to SCLC patients, in what population and the impact of the type of ICIs during rechallenge therapy on the prognosis of patients.

## Materials and methods

### Data collection

This study meets the requirements of Declaration of Helsinki (as revised in 2013). As this was a retrospective study, patient consent was not required. The ES-SCLC patients receiving PD-1/PD-L1 inhibitors plus chemotherapy in the Chinese People’s Liberation Army (PLA) General Hospital, Chinese PLA 304 Hospital and Chinese PLA 307 Hospital from January 2017 to October 2021 were retrospectively collected. The inclusion criteria were as follows (Bray et al., 2018): patients were diagnosed with SCLC; (Oronsky et al., 2017) patients received PD-1/PD-L1 inhibitors plus chemotherapy and already had progressive disease; (Früh et al., 2013) the initial immunotherapy included at least two cycles of PD-1/PD-L1 inhibitors; (Rudin et al., 2015) the treatment record was complete beyond first progression; (Bernhardt and Jalal, 2016) patients had at least one tumor evaluation before and beyond first progression. The exclusion criteria were as follows: (Bray et al., 2018) patients had no tumor evaluation; (Oronsky et al., 2017) patients died within 1 month after the treatment beyond first progression; (Früh et al., 2013) patients had other primary tumor types.

Patients who received PD-1/PD-L1 inhibitors for ≥6 weeks after progressive disease (PD) were defined as RIBP, while those who received <6 weeks of PD-1/PD-L1 inhibitors beyond first progression were defined as DIBP. The clinical data included age, gender, smoking history, initial therapeutic schedule, Eastern Cooperative Oncology Group performance status (ECOG PS), brain/liver/bone metastases, best response to initial immunotherapy, type of RECIST1.1-defined progression and treatment except ICIs beyond progression.

### Efficacy evaluation

In terms of short-term efficacy, tumor response, including complete response (CR), partial response (PR), stable disease (SD) and PD, was assessed according to the RECIST1.1 ((Eisenhauer et al., 2009)). The primary endpoint was PFS, which was defined as the period from the initiation of the post-PD treatment to disease progression or death from any cause. The secondary endpoints were OS, objective response rate (ORR), and disease control rate (DCR). OS was defined as the period from the initiation of the post-PD treatment to death from any cause. The ORR was defined as the proportion of CR and PR, while the DCR was the proportion of CR, PR, and SD. The durable clinical benefit (DCB) was defined as the best response of CR/PR or SD lasting ≥6 months. No durable benefit (NDB) was defined as the best response of PD or SD lasting <6 months. The date of the last follow-up was 25 January 2022.

### Statistical analysis

Categorical variables were compared using Chi-square or Fisher’s exact test. Continuous or ordinal variables were compared using the Student’s t-test or Mann-Whitney U test. Inverse propensity score weighting (IPSW) method was used with “WeightIt” R package to control the differences of baseline clinical characteristics to avoid the interference of other factors. As for the survival data before weighting and after weighting, Kaplan-Meier method and Log-Rank test were used with “survival” R package to compare the differences of PFS and OS of the patients in the two groups. Cox proportional-hazards regression was performed to calculate the hazard ratios (HRs) and the 95% confidence interval (CI). *p*-values were calculated based on a two-sided assumption, and *p* < 0.05 was considered to be statistically significant. Statistical analyses were performed using R (version 3.6.3) for the IPSW method and SPSS 22.0 for other analyses.

## Results

### Patient clinical characteristics

There were 506 SCLC patients who received PD-1/PD-L1 inhibitors, of which 100 patients met the inclusion criteria of this study. There were 45 patients in the RIBP group and 55 patients in the DIBP group. The starting point of this study was the initiation of the treatment beyond first progression after initial immunotherapy. The baseline clinical characteristics of the two groups were summarized in Table 1. The median age of the total population was 61 (range, 32–80) years. 85 (85.0%) patients were male, and 71 (71.0%) patients had a smoking history. All patients had an extensive disease. 40.0% patients had brain metastasis, 37.0% had liver metastasis, and 39.0% had bone metastasis. Most patients (93.0%) had an ECOG PS of 0–1. Most clinical features except gender were well balanced between the two groups. Compared with the RIBP group, there were more men in the DIBP group with a statistically significant difference.

**TABLE 1:** Baseline characteristics of the patients.

Characteristics	No. of patients (%)			*p*-value
	All patients (n = 100)	RIBP group (n = 45)	DIBP group (n = 55)	
Age				
Median age (range), years	61 (32–80)	61 (32–79)	59 (43–80)	0.552
<60	48 (48.0%)	20 (44.4%)	28 (50.9%)	
≥60	52 (52.0%)	25 (55.6%)	27 (49.1%)	
Sex				0.004
Male	85 (85.0%)	33 (73.3%)	52 (94.5%)	
Female	15 (15.0%)	12 (26.7%)	3 (5.5%)	
Smoking history				0.507
Ever	71 (71.0%)	30 (66.7%)	41 (74.5%)	
Never	29 (29.0%)	15 (33.3%)	14 (25.5%)	
Lines of previous immunotherapy				0.690
1	53 (53.0%)	25 (55.6%)	28 (50.9%)	
≥2	47 (47.0%)	20 (44.4%)	27 (49.1%)	
ICI type in previous line				0.067
PD-1 inhibitor	74 (74.0%)	29 (64.4%)	45 (81.8%)	
PD-L1 inhibitor	26 (26.0%)	16 (35.6%)	10 (18.2%)	
Best response to previous line				0.438
PR	46 (46.0%)	23 (51.1%)	23 (41.8%)	
SD	28 (28.0%)	11 (24.4%)	17 (30.9%)	
PD	26 (26.0%)	11 (24.4%)	15 (27.3%)	
Brain metastasis				0.305
Yes	40 (40.0%)	15 (33.3%)	25 (45.5%)	
No	60 (60.0%)	30 (66.7%)	30 (54.5%)	
Liver metastasis				0.837
Yes	37 (37.0%)	16 (35.6%)	21 (38.2%)	
No	63 (63.0%)	29 (64.4%)	34 (61.8%)	
Bone metastasis				0.840
Yes	39 (39.0%)	17 (37.8%)	22 (40.0%)	
No	61 (61.0%)	28 (62.2%)	33 (60.0%)	
ECOG PS				1.000
0–1	93 (93.0%)	42 (93.3%)	51 (92.7%)	
2	7 (7.0%)	3 (6.7%)	4 (7.3%)	
The type of first progression				0.386
Target leisions	66 (66.0%)	29 (64.4%)	37 (67.3%)	
New leisions	15 (15.0%)	9 (20.0%)	6 (10.9%)	
Both	19 (19.0%)	7 (15.6%)	12 (21.8%)	
Treatment regimens beyond first progression				0.762
Chemotherapy with/without ICIs	65 (65.0%)	31 (68.9%)	34 (61.8%)	
Anti-angiogenesis therapy with/without ICIs	20 (20.0%)	8 (17.8%)	12 (21.8%)	
Chemotherapy plus anti-angiogenesis therapy with/without ICIs	15 (15.0%)	6 (13.3%)	9 (16.4%)	

Abbreviations: ICI, immune checkpoint inhibitor; PD-1, programmed cell death-1; PD-L1, programmed cell death-ligand 1; PR, partial response; SD, steady disease; PD, progressive disease; ECOG PS, Eastern Cooperative Oncology Group Performance Status; RIBP, rechallenge of immunotherapy beyond progression; DIBP, discontinuation of immunotherapy beyond progression.

Prior to first progression, 29 (64.4%) patients received PD-1 inhibitors (nivolumab, pembrolizumab or sintilimab), and 16 (35.6%) patients received PD-L1 inhibitors (atezolizumab or durvalumab) in the RIBP group. 45 (81.8%) patients received PD-1 inhibitors, and 10 (18.2%) patients received PD-L1 inhibitors in the DIBP group. Before first progression, 46 (46.0%) patients had PR as their best response, 28 (28.0%) patients had SD, 26 (26.0%) patients had PD. 66.0% patients experienced the first progression due to the progression of target lesions, 15.0% patients due to the presence of new lesions, and 19.0% patients due to both the progression of target lesions and the presence of new lesions. In the RIBP group, 31 (68.9%) patients received ICIs combined with chemotherapy, 8 (17.8%) patients received ICIs combined with anti-angiogenesis therapy, and 6 (13.3%) patients received ICIs combined with chemotherapy plus anti-angiogenesis therapy following first progression. In the DIBP group, 34 (61.8%) patients received chemotherapy, 12 (21.8%) patients received anti-angiogenesis therapy, and 9 (16.4%) patients received chemotherapy plus anti-angiogenesis therapy following first progression. Up to the follow-up date, 85 (85.0%) patients experienced the second progression, including 37 (82.2%) patients in the RIBP group and 48 (87.3%) patients in the DIBP group. 53 (53.0%) patients died, including 17 (37.8%) patients in the RIBP group and 36 (65.5%) patients in the DIBP group.

### Efficacy beyond first progression

IPSW method was performed to balance the distribution of covariates by minimizing the standardized mean difference in the RIBP and DIBP groups in the total population (Supplementary Table S1). After weighting, the ORR in the RIBP group was not statistically different from that in the DIBP group (29.9% vs. 13.6%, *p* = 0.118). The DCR was significantly higher in the RIBP group than that in the DIBP group (79.7%% vs. 55.7%, *p* = 0.027). The proportion of DCB was significantly higher in the RIBP group (40.7 vs. 20.7%, *p* = 0.025, Table 2).

**TABLE 2:** Short-term effect in total population.

Short-term effect	Before weighting			After weighting		
	RIBP group (*n* = 45)	DIBP group (*n* = 55)	*p*-value	RIBP group (%)	DIBP group (%)	*p*-value
Best response, n (%)						
CR	0	0				
PR	12 (26.7%)	7 (12.7%)		29.9	13.6	
SD	24 (53.3%)	22 (40.0%)		49.8	42.1	
PD	9 (20.0%)	26 (47.3%)		20.3	44.3	
ORR	26.7%	12.7%	0.123	29.9	13.6	0.118
DCR	80.0%	52.7%	0.006	79.7	55.7	0.027
Clinical benefit, n (%)			0.037			0.025
DCB	17 (37.8%)	10 (18.2%)		40.7	20.7	
NDB	24 (53.3%)	41 (74.5%)		45.1	72.1	
Not available	4 (8.9%)	4 (7.3%)		14.2	7.2	

Abbreviations: CR, complete response; PR, partial response; SD, steady disease; PD, progressive disease; ORR, objective response rate; DCR, disease control rate; DCB, durable clinical benefit; NDB, no durable benefit; RIBP, rechallenge of immunotherapy beyond progression; DIBP, discontinuation of immunotherapy beyond progression.

By the exploratory subgroup analysis, unweighted for covariates between the RIBP and DIBP groups, RIBP showed a significant benefit in terms of OS and PFS in the overall population and particularly for OS in males, first-line initial immunotherapy, initial PD-L1 inhibitors, SD/PD response to initial immunotherapy, no brain or liver metastases and ECOG 0–1 subgroups (Supplementary Figures S1, S2).

**FIGURE 1:**
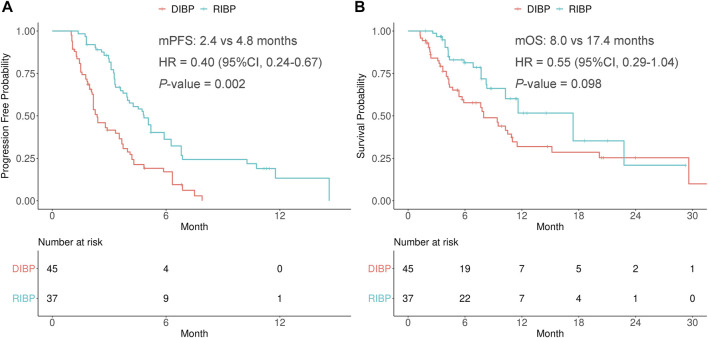
Kaplan-Meier curves of PFS **(A)** and OS **(B)** from weighted data in the total study population. RIBP, rechallenge of immunotherapy beyond progression; DIBP, discontinuation of immunotherapy beyond progression; mPFS, median progression-free survival; mOS, median overall survival; HR, hazard ratio; CI, confidence interval.

After weighting, PFS was statistically significantly longer for patients in the RIBP group than for those in the DIBP group in the total population (mPFS: 4.8 vs. 2.4 months; HR, 0.40; 95%CI: 0.24–0.67; *p* = 0.002). The median OS in the RIBP group was longer than that in the DIBP group (mOS, 17.4 vs. 8.0 mon; HR, 0.55; 95%CI: 0.29–1.04), although the difference was not statistically significant (*p* = 0.098, Figure 1).

### Subgroup analysis by the lines of previous immunotherapy

A subgroup analysis was conducted to explore the efficacy of RIBP based on the lines of previous immunotherapy (first-line and second-line or later). IPSW method was performed to balance the distribution of covariates by minimizing the standardized mean difference of RIBP group and DIBP group in the first-line and second-line or later initial immunotherapy subgroups (Supplementary Tables S2).

**FIGURE 2:**
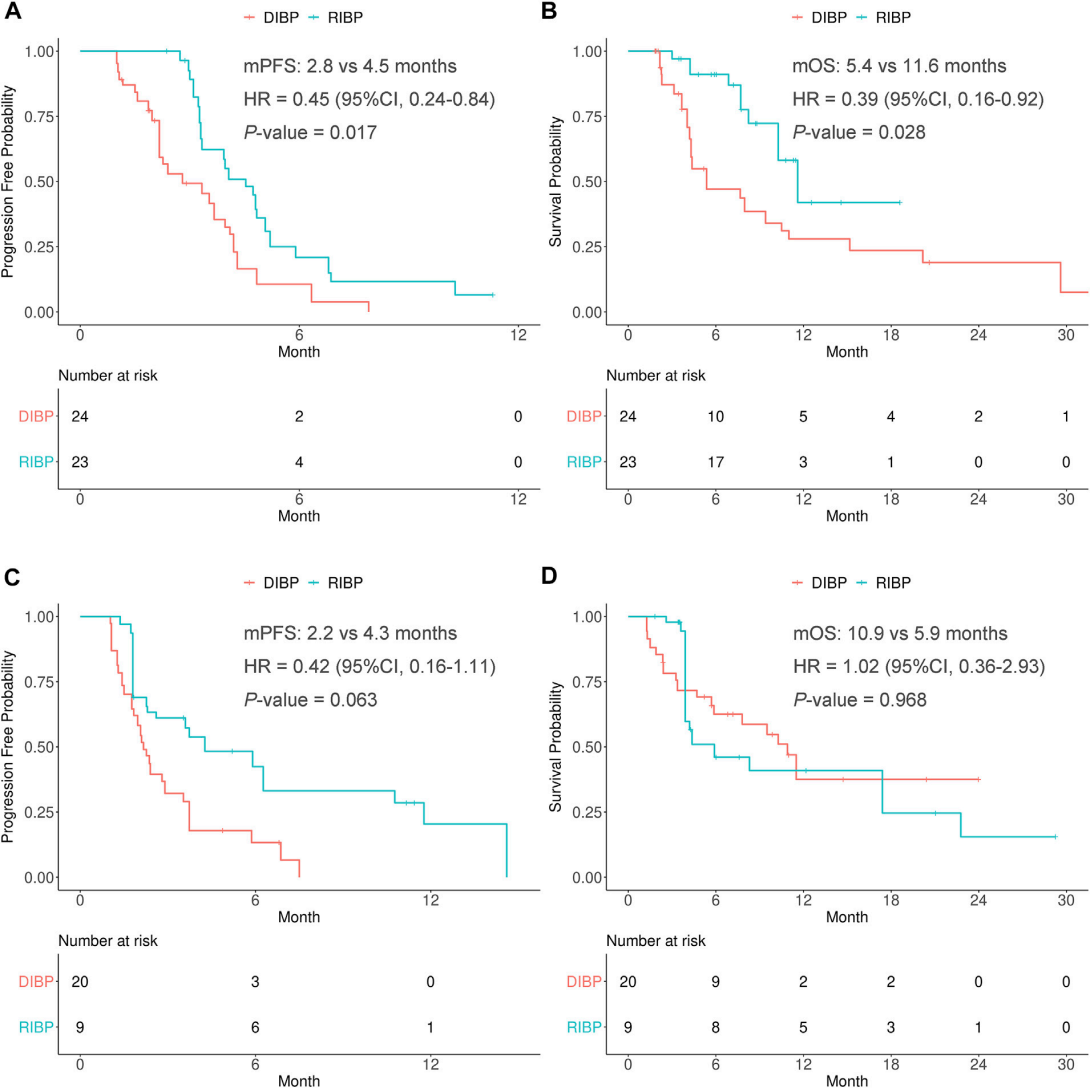
Kaplan-Meier curves of PFS **(A,C)** and OS **(B,D)** from weighted data in first-line and second-line or later subgroups. RIBP, rechallenge of immunotherapy beyond progression; DIBP, discontinuation of immunotherapy beyond progression; mPFS, median progression-free survival; mOS, median overall survival; HR, hazard ratio; CI, confidence interval.

In the weighted first-line initial immunotherapy subgroup, the median PFS was statistically significantly different between the RIBP and DIBP groups (mPFS: 4.5 vs. 2.8 mon; HR, 0.45; 95% CI: 0.24–0.84; *p* = 0.017), and OS showed similar results (mOS: 11.6 vs. 5.4 months; HR, 0.39; 95% CI: 0.16–0.92; *p* = 0.028). The Kaplan-Meier curves of the weighted first-line initial immunotherapy subgroup were shown in Figures 2A,B.

There were no statistically significant differences in the PFS and OS between RIBP group and DIBP group in the weighted second-line or later initial immunotherapy subgroup (mPFS: 4.3 vs. 2.2 months, HR: 0.42, 95% CI: 0.16–1.11, *p* = 0.063; mOS: 5.9 vs. 10.9 months, HR: 1.02, 95% CI: 0.36–2.93, *p* = 0.968). The Kaplan-Meier curves of the weighted second-line or later initial immunotherapy subgroup were shown in Figures 2C,D.

### Subgroup analysis by the best response to initial immunotherapy

A subgroup analysis was conducted to explore the efficacy of RIBP based on the best response to initial immunotherapy (the PR response and SD/PD response). IPSW method was performed to balance the distribution of covariates by minimizing the standardized mean difference of RIBP group and DIBP group in the PR response and SD/PD response to initial immunotherapy subgroups (Supplementary Tables S3).

**FIGURE 3:**
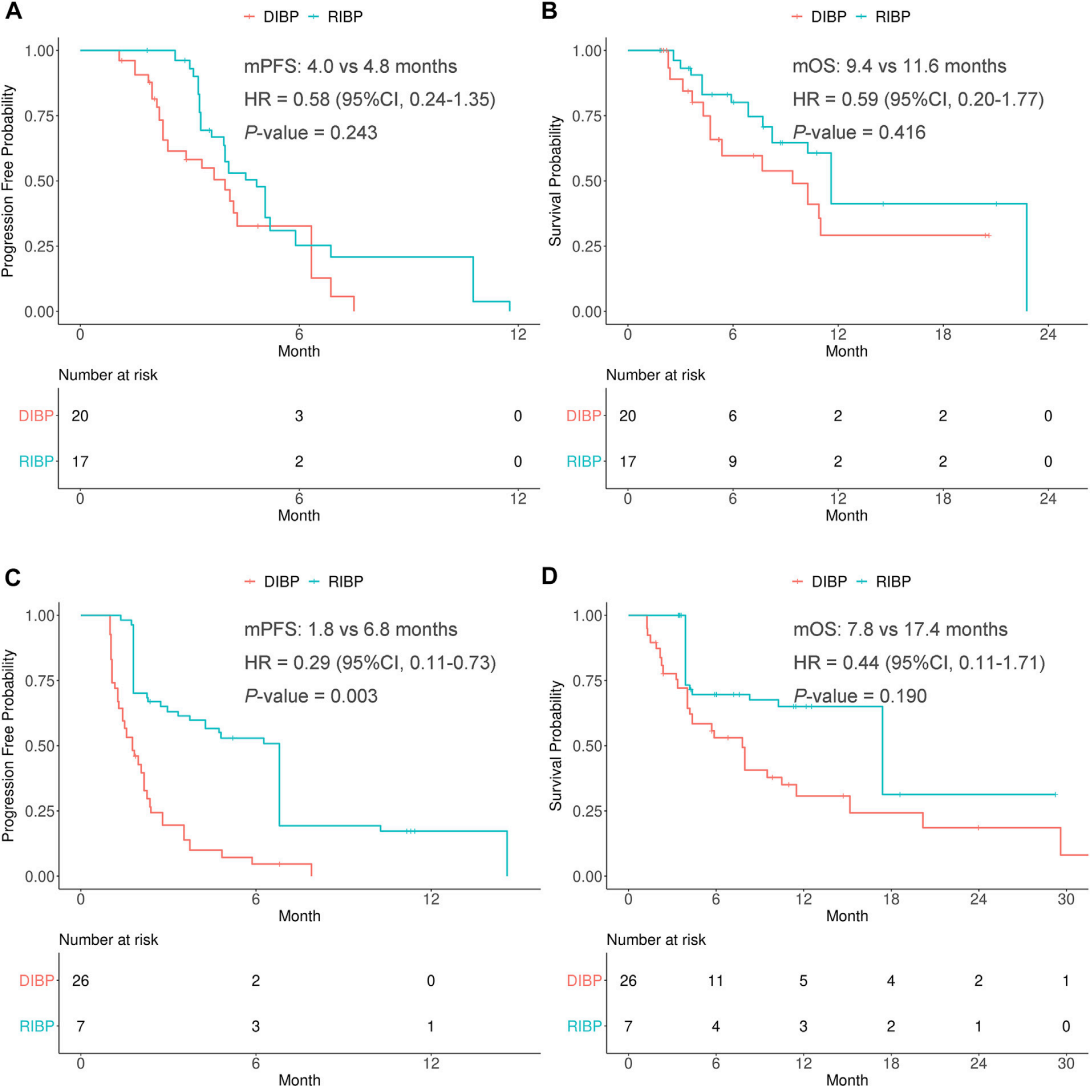
Kaplan-Meier curves of PFS **(A,C)** and OS **(B,D)** from weighted data in the PR response and SD/PD response to initial immunotherapy subgroups. RIBP, rechallenge of immunotherapy beyond progression; DIBP, discontinuation of immunotherapy beyond progression; mPFS, median progression-free survival; mOS, median overall survival; HR, hazard ratio; CI, confidence interval.

In the weighted PR response to initial immunotherapy subgroup, there were no statistically significant differences in the PFS and OS between RIBP group and DIBP group (mPFS: 4.8 vs. 4.0 months, HR: 0.58, 95% CI: 0.24–1.35, *p* = 0.243; mOS: 11.6 vs. 9.4 months, HR: 0.59, 95% CI: 0.20–1.77, *p* = 0.416). The Kaplan-Meier curves of the weighted PR response to initial immunotherapy subgroup were shown in Figures 3A,B.

In the weighted SD/PD response to initial immunotherapy subgroup, the median PFS was statistically significantly different between the RIBP and DIBP groups (mPFS: 6.8 vs. 1.8 months; HR, 0.29; 95% CI: 0.11–0.73; *p* = 0.003). The median OS in the RIBP group was longer than that in the DIBP group (mOS: 17.4 vs. 7.8 months; HR, 0.44; 95%CI: 0.11–1.71), although the difference was not statistically significant (*p* = 0.190). The Kaplan-Meier curves of the weighted SD/PD response to initial immunotherapy subgroup were shown in Figures 3C,D.

### Subgroup analysis by the treatment strategy beyond first progression

The patients who received chemotherapy with or without ICIs were defined as the chemotherapy group, while the patients who received anti-angiogenesis therapy with or without ICIs were defined as the anti-angiogenesis therapy group. IPSW method was performed to balance the distribution of covariates by minimizing the standardized mean difference of RIBP group and DIBP group in the chemotherapy and anti-angiogenesis therapy subgroups (Supplementary Tables S4).

**FIGURE 4:**
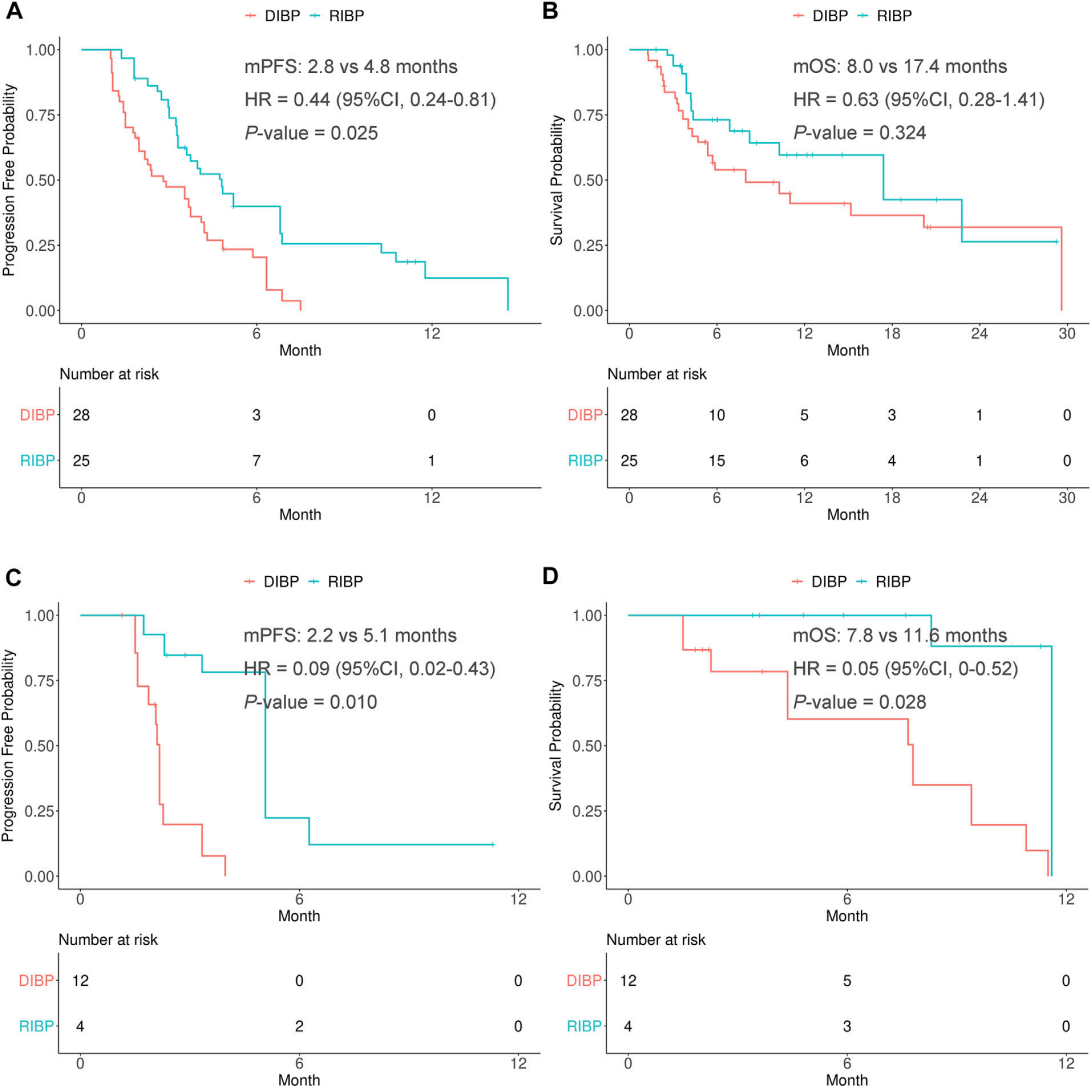
Kaplan-Meier curves of PFS **(A,C)** and OS **(B,D)** from weighted data in the chemotherapy and anti-angiogenesis therapy subgroups. RIBP, rechallenge of immunotherapy beyond progression; DIBP, discontinuation of immunotherapy beyond progression; mPFS, median progression-free survival; mOS, median overall survival; HR, hazard ratio; CI, confidence interval.

In the weighted chemotherapy subgroup, the median PFS was statistically significantly different between the RIBP and DIBP groups (mPFS: 4.8 vs. 2.8 months; HR, 0.44; 95% CI: 0.24–0.81; *p* = 0.025). The median OS in the RIBP group was longer than that in the DIBP group (mOS, 17.4 vs. 8.0 months; HR, 0.63; 95%CI: 0.28–1.41), although the difference was not statistically significant (*p* = 0.324). The Kaplan-Meier curves of the weighted chemotherapy subgroup were shown in Figures 4A,B.

In the weighted anti-angiogenesis therapy subgroup, there were statistically significant differences in the PFS and OS between RIBP group and DIBP group (mPFS: 5.1 vs. 2.2 months, HR: 0.09, 95% CI: 0.02–0.43, *p* = 0.010; mOS: 11.6 vs. 7.8 months, HR: 0.05, 95%CI: 0.004–0.52, *p* = 0.028). The Kaplan-Meier curves of the weighted anti-angiogenesis therapy subgroup were shown in Figures 4C,D.

## Discussion

“Tumor flare” has sometimes been associated with the application of immune checkpoint inhibitors, which refers to the transient progression in target tumor lesions or the occurrence of new lesions prior to clinical responses in patients receiving immunotherapy (Chiou and Burotto, 2015; Hodi et al., 2016; Seymour et al., 2017). The two main reasons resulting in “tumor flare” may be transient immune cell infiltration into the tumor and the delayed antitumor response (Finke et al., 2007). Therefore, “tumor flare” occurring with immunotherapy will be evaluated as disease progression and may lead to discontinuation of immunotherapy following RECIST1.1 criteria that precedes the fully realized clinical benefit (Chiou and Burotto, 2015). Thus, the rechallenge of immunotherapy holds the theoretical potential to bring benefits to patients with cancer. In fact, several clinical trials have demonstrated the effect of RIBP in advanced renal cell carcinoma, squamous cell carcinoma of the head and neck, melanoma, NSCLC, and urothelial carcinoma. However, it is unclear whether the RIBP could bring benefits to SCLC patients. Therefore, our study aims to evaluate the effect of the RIBP in SCLC patients.

In this study, only 100 SCLC patients who received PD-1/PD-L1 inhibitors in three medical centers were screened for the final analysis. Patients who did not receive treatment or went to other hospitals for treatment beyond first progression were excluded. As this was a retrospective study, there would be a bias in the therapeutic schedule of patients who are followed up by telephone. Therefore, our study only included patients who had complete medical records in our hospitals beyond first progression. The previous studies of RIBP usually compared the clinical response or survival time from the start of treatment beyond first progression to the second progression between RIBP and DIBP groups (Hanovich et al., 2020). Thus, the related analyses were performed in our study.

The IPSW method was used to control the difference in the baseline clinical characteristics of the RIBP and DIBP groups within a certain range. Thus, the influence of other potential interference factors was excluded by weighting. In terms of short-term efficacy, there was no statistically significant difference in the ORR of the RIBP and DIBP groups. However, there were significant differences in the DCR and the proportion of DCB between the two groups after weighting, indicating that RIBP may benefit SCLC patients. In terms of long-term efficacy, the rechallenge with PD-1/PD-L1 inhibitors could prolong the PFS of SCLC patients, but the OS of the RIBP group in the total population was not significantly prolonged.

We conducted subgroup analysis according to the lines of previous immunotherapy. There were statistically significant differences in PFS and OS between the RIBP and DIBP groups in the weighted first-line subgroup. However, there was no significant difference in PFS and OS between the two groups in the weighted second-line or later subgroup. The possible reason was that patients in the first-line subgroup generally had relatively adequate immune reserves, while patients in the second-line or later subgroup had poor immune reserves. Additionally, the proportion of SCLC patients who benefited from RIBP was small, resulting possible bias in population selection. Therefore, the conclusions drawn by our study still needs to be verified by large prospective clinical trials.

A subgroup analysis by the best response to initial immunotherapy was then performed. We found that there was no significant difference in PFS and OS between the RIBP group and the DIBP group in the weighted PR response subgroup. There was a statistically significant difference in PFS between the two groups in the weighted SD/PD response subgroup, but there was no statistically significant difference in OS. Some early clinical studies only allowed patients with response of CR/PR or SD lasting ≥3 months in initial immunotherapy to receive the rechallenge of immunotherapy (Lebbé et al., 2014; Jansen et al., 2019). However, in a retrospective study, 6 of 26 patients with renal cell carcinoma initially unresponsive to immunotherapy responded to the rechallenge of ICI therapy (Abou Alaiwi et al., 2020). In a subgroup analysis of the phase III CheckMate 025 clinical trial, 153 patients received the retreatment of immunotherapy, 142 of which were evaluable for response beyond first progression. 12 of 113 patients with an initial best response of SD/PD had a tumor reduction ≥30% (Escudier et al., 2017), suggesting the patients in the SD/PD response subgroup could still benefit from the rechallenge of immunotherapy. In our study, the PFS of RIBP group was significantly better than that of the DIBP group with a statistically significant difference in the SD/PD response subgroup.

Although the treatment regimens before first progression were all PD-1/PD-L1 inhibitors plus chemotherapy in this study, the treatment regimens in the RIBP group beyond first progression included ICIs plus chemotherapy, ICIs plus anti-angiogenesis therapy and ICIs plus chemotherapy and anti-angiogenesis therapy, while the treatment regimens in the DIBP group beyond first progression included chemotherapy alone, anti-angiogenesis therapy alone and chemotherapy plus anti-angiogenesis therapy. To address the differences in treatment regimens beyond progression, we divided the total population of patients into three groups based on treatment regimens after progression, including chemotherapy group, anti-angiogenesis therapy group, and chemotherapy plus anti-angiogenesis therapy group. The IPSW method was used to control the differences in the treatment regimens and other clinical characteristics of the RIBP and DIBP groups. In addition, we performed a subgroup analysis according to the treatment regimen beyond first progression. Finally, we found that in the weighted chemotherapy subgroup, there was a statistically significant difference in PFS but no difference in OS between the RIBP and DIBP groups. There were statistically significant differences in PFS and OS between the two groups in the anti-angiogenesis therapy group. Due to the small number of patients in the chemotherapy plus anti-angiogenesis therapy group, the analysis of this part was discarded. Taken together, no matter patients chose to switch to other chemotherapy or anti-angiogenesis therapy beyond first progression, the addition of PD-1/PD-L1 inhibitors could benefit SCLC patients. By the way, the studies of RIBP in the real world were slightly different from those in the clinical trials, as patients received immunotherapy combined with other chemotherapy or anti-angiogenesis therapy beyond first progression in the real world, which would not affect the possibility of patients benefiting from other chemotherapy drugs or anti-angiogenesis therapy, while the clinical trials of RIBP generally required patients to continue to receive immunotherapy alone (George et al., 2016; Gandara et al., 2018b). Thus, our subgroup analysis of post-progression treatment regimens was necessary.

There were three potential reasons for the effectiveness of RIBP in this study. Firstly, patients received chemotherapy and/or anti-angiogenesis therapy beyond first progression. Patients may respond to these drugs, and these drugs may change the tumor immune microenvironment. Therefore, RIBP might bring benefit to these SCLC patients. Our study has excluded the influence of post-progression treatment regimens by IPSW method and subgroup analysis. Secondly, there is atypical response in immunotherapy, and initially evaluated disease progression by RECIST1.1 may not be true progression, which was called pseudoprogression. However, the incidence of pseudoprogression is rather low (usually 1.5–4%). Therefore, it could only explain the benefit of a small proportion of these patients. Thirdly, the priming of the immune system for an antitumor response needs some time, resulting a delayed immune response (Kuczynski et al., 2013; Robert et al., 2013).

We found and demonstrated that the rechallenge of PD-1/PD-L1 inhibitors could benefit SCLC patients, but there were still limitations of our study. Firstly, this was a retrospective study, and some confounding factors and selective bias could not be avoided. Secondly, irRECIST criteria was not used in the real world. Thus, there were some patients experiencing pseudoprogression in our study. However, the incidence of pseudoprogression is rather low (Chiou and Burotto, 2015; Won et al., 2020). Thirdly, the cutoff value of 6 weeks might not be optimal. Lastly, no sufficient data for biomarker was obtained in this study, so we did not analyze the biomarkers for identifying patients more likely to benefit from RIBP. We hope to expand samples to explore the biomarker for predicting the effect of RIBP in the future.

In conclusion, rechallenge of immunotherapy could benefit patients with SCLC, and the discontinuation of immunotherapy beyond first progression may be premature, especially in the first-line initial immunotherapy subgroup or SD/PD response to initial immunotherapy subgroup.

## Data Availability

The raw data supporting the conclusions of this article will be made available by the authors, without undue reservation.
