# Supplementary material for: Rechallenge of immunotherapy beyond progression in patients with extensive-stage small-cell lung cancer
Source: Front Pharmacol. 2022 Sep 6;13:967559. doi: 10.3389/fphar.2022.967559 (PMC9485935; doi:10.3389/fphar.2022.967559)
Supplement: Supplementary file 1 [file DataSheet1.ZIP › Suppl.data/Table S2.docx]

Table S2 Summary of the baseline characteristics of patients in the subgroups by lines of previous immunotherapy after weighting

| Characteristics | First-line group | | | Second-line or later group | | | |
| --- | --- | --- | --- | --- | --- | --- | --- |
|  | DIBP group | RIBP group | Standard Mean Diff. | | DIBP group | RIBP group | Standard Mean Diff. |
| Age | 59.91 | 60.74 | 0.08 | | 59.37 | 60.83 | 0.14 |
| Sex |  |  |  | |  |  |  |
| Male | 0.89 | 0.86 | 0.03 | | 0.90 | 0.83 | 0.07 |
| Female | 0.11 | 0.14 | 0.03 | | 0.10 | 0.17 | 0.07 |
| Smoking history |  |  |  | |  |  |  |
| Ever | 0.76 | 0.79 | 0.03 | | 0.73 | 0.57 | 0.16 |
| Never | 0.24 | 0.21 | 0.03 | | 0.27 | 0.43 | 0.16 |
| ECOG PS |  |  |  | |  |  |  |
| 0-1 | 0.98 | 1.00 | 0.02 | | 0.90 | 0.92 | 0.02 |
| 2 | 0.02 | 0.00 | 0.02 | | 0.10 | 0.08 | 0.02 |
| Metastatic sites |  |  |  | |  |  |  |
| Bone metastasis | 0.39 | 0.40 | 0.01 | | 0.39 | 0.58 | 0.19 |
| Brain metastasis | 0.32 | 0.31 | 0.01 | | 0.64 | 0.65 | 0.01 |
| Liver metastasis | 0.28 | 0.27 | 0.01 | | 0.41 | 0.27 | 0.14 |
| Treatment regimens  beyond first progression |  |  |  | |  |  |  |
| Chemotherapy  with/without ICIs | 0.55 | 0.59 | 0.04 | | 0.76 | 0.84 | 0.08 |
| Anti-angiogenesis therapy with/without ICIs | 0.30 | 0.26 | 0.04 | | 0.13 | 0.10 | 0.03 |
| Chemotherapy plus  anti-angiogenesis therapy with/without ICIs | 0.15 | 0.15 | 0.00 | | 0.11 | 0.06 | 0.05 |
| The type of first progression |  |  |  | |  |  |  |
| New leisions | 0.12 | 0.12 | 0.00 | | 0.18 | 0.17 | 0.01 |
| Target leisions | 0.64 | 0.67 | 0.03 | | 0.65 | 0.76 | 0.11 |
| Both | 0.24 | 0.21 | 0.03 | | 0.16 | 0.07 | 0.09 |
| ICI type in previous line |  |  |  | |  |  |  |
| PD-1 inhibitor | 0.61 | 0.56 | 0.05 | | 0.89 | 0.90 | 0.01 |
| PD-L1 inhibitor | 0.39 | 0.44 | 0.05 | | 0.11 | 0.10 | 0.01 |
| Best response to previous line |  |  |  | |  |  |  |
| PR | 0.61 | 0.61 | 0.00 | | 0.26 | 0.37 | 0.11 |
| SD/PD | 0.39 | 0.39 | 0.00 | | 0.74 | 0.63 | 0.11 |

Abbreviations: ICI, immune checkpoint inhibitor; PD-1, programmed cell death-1; PD-L1, programmed cell death-ligand 1; PR, partial response; SD, steady disease; PD, progressive disease; ECOG PS, Eastern Cooperative Oncology Group Performance Status; RIBP, rechallenge of immunotherapy beyond progression; DIBP, discontinuation of immunotherapy beyond progression.
