# Supplementary material for: Rechallenge of immunotherapy beyond progression in patients with extensive-stage small-cell lung cancer
Source: Front Pharmacol. 2022 Sep 6;13:967559. doi: 10.3389/fphar.2022.967559 (PMC9485935; doi:10.3389/fphar.2022.967559)
Supplement: Supplementary file 1 [file DataSheet1.ZIP › Suppl.data/Table S3.docx]

Table S3 Summary of the baseline characteristics of patients in the subgroups by best response to previous line after weighting

| Characteristics | PR response group | | | | SD/PD response group | | |
| --- | --- | --- | --- | --- | --- | --- | --- |
|  | DIBP group | RIBP group | Standard Mean Diff. | DIBP group | | RIBP group | Standard Mean Diff. |
| Age | 59.48 | 59.96 | 0.05 | 59.85 | | 59.57 | 0.03 |
| Sex |  |  |  |  | |  |  |
| Male | 0.84 | 0.81 | 0.03 | 0.91 | | 0.91 | 0.00 |
| Female | 0.16 | 0.19 | 0.03 | 0.09 | | 0.09 | 0.00 |
| Smoking history |  |  |  |  | |  |  |
| Ever | 0.55 | 0.55 | 0.00 | 0.83 | | 0.87 | 0.04 |
| Never | 0.45 | 0.45 | 0.00 | 0.17 | | 0.13 | 0.04 |
| ECOG PS |  |  |  |  | |  |  |
| 0-1 | 1.00 | 0.96 | 0.04 | 0.90 | | 0.97 | 0.07 |
| 2 | 0.00 | 0.04 | 0.04 | 0.10 | | 0.03 | 0.07 |
| Metastatic sites |  |  |  |  | |  |  |
| Bone metastasis | 0.33 | 0.37 | 0.04 | 0.40 | | 0.64 | 0.24 |
| Brain metastasis | 0.37 | 0.36 | 0.01 | 0.50 | | 0.64 | 0.14 |
| Liver metastasis | 0.30 | 0.35 | 0.05 | 0.45 | | 0.47 | 0.02 |
| Lines of previous immunotherapy |  |  |  |  | |  |  |
| 1 | 0.71 | 0.66 | 0.05 | 0.40 | | 0.46 | 0.06 |
| ≥2 | 0.29 | 0.34 | 0.05 | 0.60 | | 0.54 | 0.06 |
| Treatment regimens  beyond first progression |  |  |  |  | |  |  |
| Chemotherapy  with/without ICIs | 0.73 | 0.73 | 0.00 | 0.67 | | 0.84 | 0.17 |
| Anti-angiogenesis therapy with/without ICIs | 0.21 | 0.15 | 0.06 | 0.15 | | 0.13 | 0.02 |
| Chemotherapy plus  anti-angiogenesis therapy with/without ICIs | 0.06 | 0.13 | 0.07 | 0.18 | | 0.03 | 0.15 |
| The type of first progression |  |  |  |  | |  |  |
| New leisions | 0.14 | 0.21 | 0.07 | 0.14 | | 0.12 | 0.02 |
| Target leisions | 0.59 | 0.58 | 0.01 | 0.73 | | 0.81 | 0.08 |
| Both | 0.26 | 0.21 | 0.05 | 0.13 | | 0.08 | 0.05 |
| ICI type in previous line |  |  |  |  | |  |  |
| PD-1 inhibitor | 0.65 | 0.64 | 0.01 | 0.89 | | 0.89 | 0.00 |
| PD-L1 inhibitor | 0.35 | 0.36 | 0.01 | 0.11 | | 0.11 | 0.00 |

Abbreviations: ICI, immune checkpoint inhibitor; PD-1, programmed cell death-1; PD-L1, programmed cell death-ligand 1; PR, partial response; SD, steady disease; PD, progressive disease; ECOG PS, Eastern Cooperative Oncology Group Performance Status; RIBP, rechallenge of immunotherapy beyond progression; DIBP, discontinuation of immunotherapy beyond progression.
