# Supplementary material for: Rechallenge of immunotherapy beyond progression in patients with extensive-stage small-cell lung cancer
Source: Front Pharmacol. 2022 Sep 6;13:967559. doi: 10.3389/fphar.2022.967559 (PMC9485935; doi:10.3389/fphar.2022.967559)
Supplement: Supplementary file 1 [file DataSheet1.ZIP › Suppl.data/Table S4.docx]

Table S4 Summary of the baseline characteristics of patients in the subgroups by treatment regimens beyond first progression after weighting

| Characteristics | Chemotherapy group | | | Anti-angiogenesis group | | |
| --- | --- | --- | --- | --- | --- | --- |
|  | DIBP group | RIBP group | Standard Mean Diff. | DIBP group | RIBP group | Standard Mean Diff. |
| Age | 60.08 | 59.64 | 0.05 | 59.62 | 59.97 | 0.03 |
| Sex |  |  |  |  |  |  |
| Male | 0.88 | 0.85 | 0.03 | 0.91 | 0.92 | 0.01 |
| Female | 0.12 | 0.15 | 0.03 | 0.09 | 0.08 | 0.01 |
| Smoking history |  |  |  |  |  |  |
| Ever | 0.69 | 0.68 | 0.01 | 0.66 | 0.86 | 0.20 |
| Never | 0.31 | 0.32 | 0.01 | 0.34 | 0.14 | 0.20 |
| ECOG PS |  |  |  |  |  |  |
| 0-1 | 0.98 | 0.98 | 0.00 | 0.94 | 1.00 | 0.06 |
| 2 | 0.02 | 0.02 | 0.00 | 0.06 | 0.00 | 0.06 |
| Metastatic sites |  |  |  |  |  |  |
| Bone metastasis | 0.38 | 0.43 | 0.05 | 0.36 | 0.65 | 0.29 |
| Brain metastasis | 0.45 | 0.46 | 0.01 | 0.50 | 0.61 | 0.11 |
| Liver metastasis | 0.39 | 0.39 | 0.00 | 0.23 | 0.14 | 0.09 |
| Lines of previous immunotherapy |  |  |  |  |  |  |
| 1 | 0.49 | 0.49 | 0.00 | 0.64 | 0.76 | 0.12 |
| ≥2 | 0.51 | 0.51 | 0.00 | 0.36 | 0.24 | 0.12 |
| The type of first progression |  |  |  |  |  |  |
| New leisions | 0.24 | 0.22 | 0.02 | 0.06 | 0.00 | 0.06 |
| Target leisions | 0.62 | 0.63 | 0.01 | 0.66 | 0.93 | 0.27 |
| Both | 0.15 | 0.15 | 0.00 | 0.27 | 0.07 | 0.20 |
| ICI type in previous line |  |  |  |  |  |  |
| PD-1 inhibitor | 0.68 | 0.71 | 0.03 | 0.92 | 0.81 | 0.11 |
| PD-L1 inhibitor | 0.32 | 0.29 | 0.03 | 0.08 | 0.19 | 0.11 |
| Best response to previous line |  |  |  |  |  |  |
| PR | 0.51 | 0.49 | 0.02 | 0.52 | 0.54 | 0.02 |
| SD/PD | 0.49 | 0.51 | 0.02 | 0.48 | 0.46 | 0.02 |

Abbreviations: ICI, immune checkpoint inhibitor; PD-1, programmed cell death-1; PD-L1, programmed cell death-ligand 1; PR, partial response; SD, steady disease; PD, progressive disease; ECOG PS, Eastern Cooperative Oncology Group Performance Status; RIBP, rechallenge of immunotherapy beyond progression; DIBP, discontinuation of immunotherapy beyond progression.
