# Supplementary figures and images for: Rechallenge of immunotherapy beyond progression in patients with extensive-stage small-cell lung cancer
Source: Front Pharmacol. 2022 Sep 6;13:967559. doi: 10.3389/fphar.2022.967559 (PMC9485935; doi:10.3389/fphar.2022.967559)

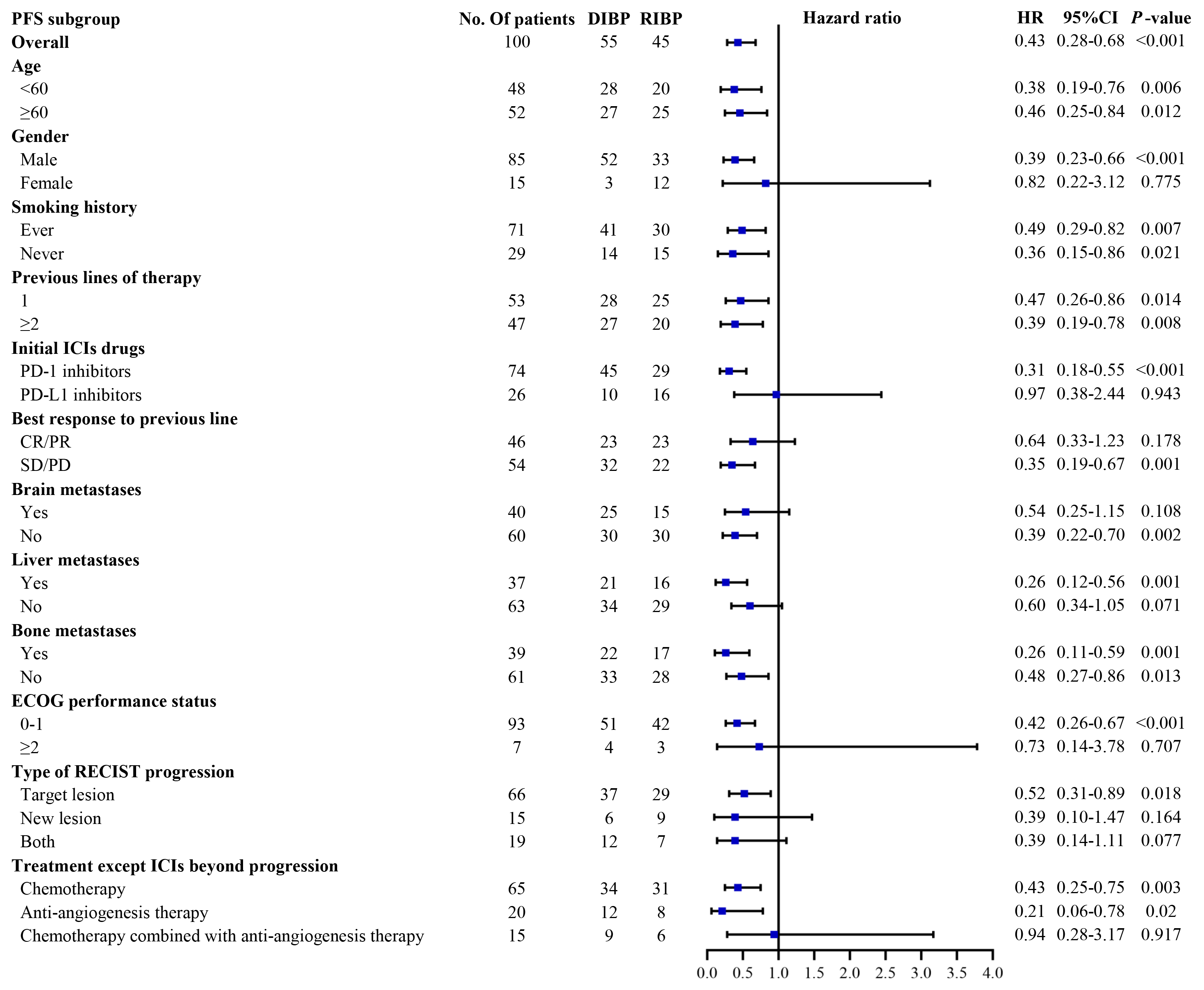

Supplement: Supplementary file 1 [file DataSheet1.ZIP › Suppl.data/Figure S1.tif]

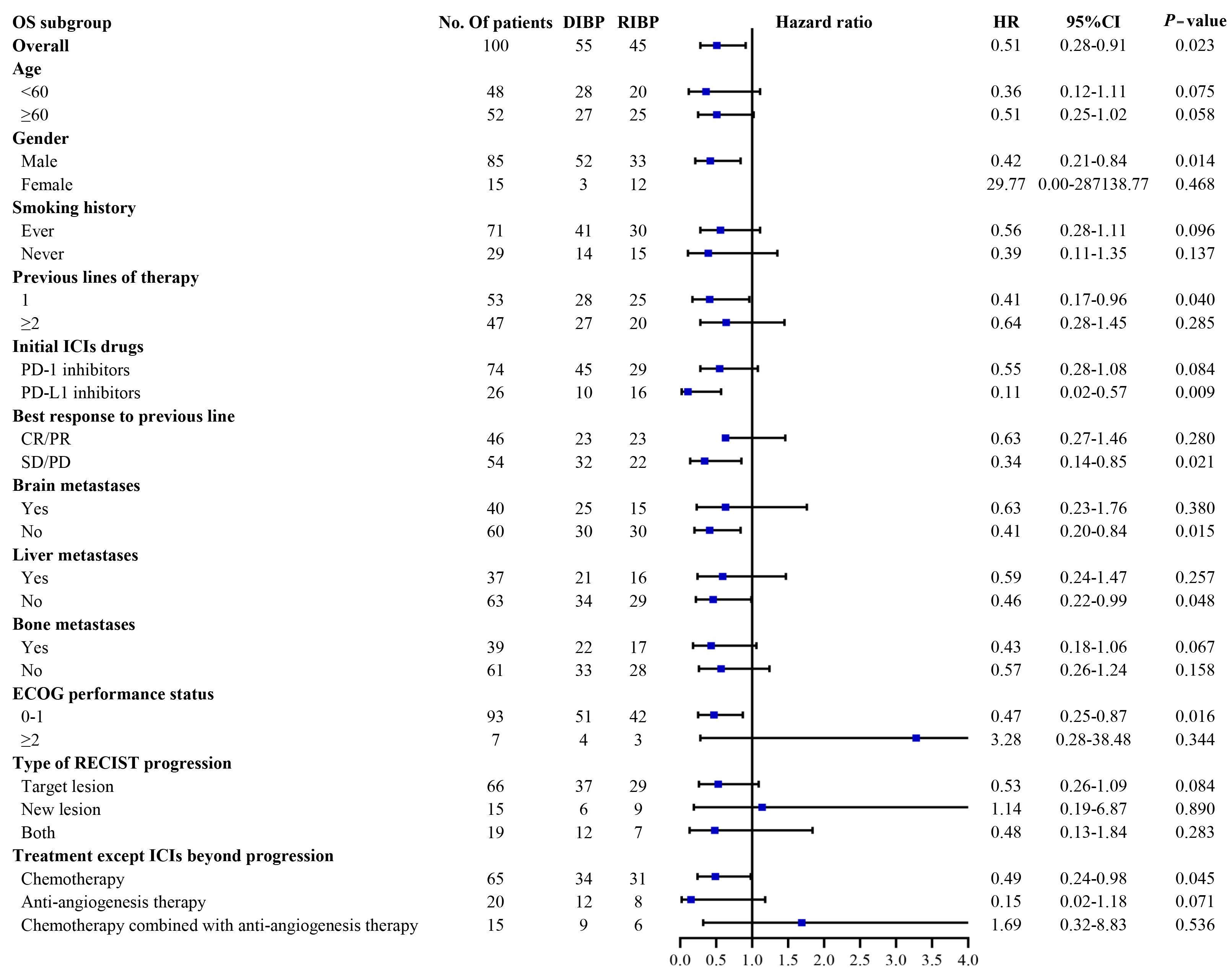

Supplement: Supplementary file 1 [file DataSheet1.ZIP › Suppl.data/Figure S2.tif]
